# Sex, PrEP, and Stigma: Experiences with HIV Pre-exposure Prophylaxis Among New York City MSM Participating in the HPTN 067/ADAPT Study

**DOI:** 10.1007/s10461-017-1964-6

**Published:** 2017-11-15

**Authors:** Julie Franks, Yael Hirsch-Moverman, Avelino S. Loquere, K. Rivet Amico, Robert M. Grant, Bonnie J. Dye, Yan Rivera, Robert Gamboa, Sharon B. Mannheimer

**Affiliations:** 1 ICAP at Columbia University, Harlem Prevention Center, 215 W. 125th St., Suite A, New York, NY 10027 USA; 2 Department of Epidemiology, Columbia University Mailman School of Public Health, New York, NY USA; 3 Department of Health Behavior and Health Education, University of Michigan, Ann Arbor, MI USA; 4 Gladstone Institutes, University of California, San Francisco, San Francisco, CA USA; 5 FHI 360, Durham, NC USA; 6 Harlem Hospital Center, New York, NY USA

**Keywords:** Pre-exposure prophylaxis, MSM, Stigma, Adherence, Sexual behavior

## Abstract

The HPTN 067/Alternative Dosing to Augment Pre-Exposure Prophylaxis Pill Taking (ADAPT) study evaluated daily and non-daily dosing schedules for oral pre-exposure prophylaxis (PrEP) to prevent HIV. A qualitative sub-study including focus groups and in-depth interviews was conducted among men who have sex with men participating in New York City to understand their experience with PrEP and study dosing schedules. The 37 sub-study participants were 68% black, 11% white, and 8% Asian; 27% were of Hispanic/Latino ethnicity. Mean age was 34 years. Themes resulting from qualitative analysis include: PrEP is a significant advance for HIV prevention; non-daily dosing of PrEP is congruent with HIV risk; and pervasive stigma connected to HIV and risk behavior is a barrier to PrEP adherence, especially for non-daily dosing schedules. The findings underscore how PrEP intersects with other HIV prevention practices and highlight the need to understand and address multidimensional stigma related to PrEP use.

## Resumen

El estudio HPTN 067/Dosificación Alternativa para Aumentar la Toma de Pastillas de la Profilaxis Pre-Exposición (conocido por su sigla en inglés, ADAPT) evaluó tres estrategias, una diaria y dos no diarias, para la dosificación de la profilaxis pre-exposición oral (PrEP) para prevenir el VIH. Se realizó un sub-estudio cualitativo que incluyó grupos focales y entrevistas en profundidad con hombres que tienen sexo con otros hombres participando en el estudio en la Ciudad de Nueva York. El motivo del sub-estudio fue entender como los participantes experimentaron la PrEP y las estrategias de dosificación del estudio. Los 37 participantes del sub-estudio fueron 68% negro, 11% blanco y 8% asiático; 27% fueron de etnia hispana/latina. La edad media fue de 34 años. Los temas que resultaron del análisis cualitativo incluyen: La PrEP es un avance significativo para la prevención del VIH; hay una congruencia entre la dosificación no diaria de PrEP y los patrones del riesgo de VIH; y el penetrante estigma relacionado con el VIH es una barrera a la adherencia a la PrEP, especialmente para las estrategias de dosificación no diarias. Los hallazgos subrayan cómo la PrEP se intersecta con otras prácticas de prevención del VIH y resaltan la necesidad de entender y abordar el estigma multidimensional relacionado con el uso de PrEP.

## Introduction

Oral emtricitabine/tenofovir disoproxil fumarate (FTC/TDF) as pre-exposure prophylaxis (PrEP) to prevent HIV acquisition was approved by the FDA in 2012 [1] and has the potential to alter the trajectory of the US epidemic if used by those at substantial risk for HIV, including men who have sex with men (MSM) [2, 3]. However, only about 4% of MSM surveyed in the 2014 National HIV Behavioral Surveillance had taken PrEP in the last 12 months, a much lower proportion than the 25% estimated to meet behavioral indications for PrEP [4]. Some potential barriers to use of the currently approved daily PrEP regimen, such as the need for long-term adherence to a daily medication [5, 6], users’ concerns about medication toxicity [7–10], and cost [9–11], may be mitigated by nondaily PrEP regimens [12–16]. Contextual factors, notably users’ perceptions and experiences of social attitudes about PrEP [17–20], can also influence its use. While perceived social norms concerning PrEP use and how PrEP fits into sexual practices have been explored hypothetically in qualitative research among people who would be potentially eligible to take PrEP [21–24], to date there is limited qualitative research exploring experiences using open-label PrEP [18, 25–27], particularly for non-daily dosing [19].

The current analysis presents findings from a qualitative sub-study conducted among New York City participants in HPTN 067/Alternative Dosing to Augment Pre-Exposure Prophylaxis Pill Taking (ADAPT), a Phase II, randomized, open-label clinical trial comparing the feasibility and acceptability of alternative PrEP dosing schedules of oral FTC/TDF. The qualitative sub-study focused on understanding contextual factors influencing participants’ use of PrEP, how participants fit PrEP into their established HIV prevention practices, and their preferences for dosing schedules.

## Methods

### Main Study Overview

Study methods and main results of HPTN 067/ADAPT have been described elsewhere [28–30]. In brief, the study assessed coverage of sexual intercourse events (defined as taking a pre-sex dose within 4 days prior to sex and taking a post-sex dose within 24 h following sex); the number of PrEP tablets required for coverage; and side effects (ClinicalTrials.gov NCT01327651). The study enrolled HIV-uninfected women in Cape Town, South Africa and HIV-uninfected MSM and transgender women (TGW) at sites in Harlem, New York City and Bangkok, Thailand. Recruitment methods varied by site. Harlem participants were recruited through a mix of community recruitment strategies, referrals from providers, word of mouth, and on-line advertising. Participants were randomly allocated 1:1:1 to one of three unblinded, open-label PrEP dosing schedules for 24 weeks of self-administered dosing: the current FDA-approved daily regimen (a single FTC/TDF tablet once a day); twice-weekly doses of a single FTC/TDF tablet with an additional tablet within 2 h after sexual intercourse (time-driven); or a single FTC/TDF tablet taken up to 48 h before sex and an additional tablet taken within 2 h after intercourse (event-driven). All participants received counseling on adherence to their assigned dosing schedule [31] and behavioral risk reduction, condoms and lubricant. Separate qualitative sub-studies including focus groups (FG) and individual in-depth interviews (IDI) were conducted at each study site to explore the social and cultural dimensions that could affect PrEP use.

### Sub-study Methods

#### Recruitment

Sub-study participants were recruited from the Harlem study sample of 179 participants. Potential participants were invited to participate in either FG or IDI after they had completed the 24 weeks of self-administered PrEP use and within 3 months of study completion. Separate FG comprised of participants from each arm were scheduled to include participants who enrolled at early and late intervals during the main study follow-up period. Similarly, IDI with participants from each arm were scheduled at early and late intervals during the main study follow-up period. Convenience sampling was used to recruit FG participants; purposeful sampling [32] was used for IDI to capture the range of experiences with self-administered PrEP, including difficulties adhering to the assigned dosing schedule or discontinuation of PrEP.

#### Data Collection

Following written informed consent, FG and IDI were conducted in English by an experienced qualitative researcher who was not involved in main study procedures, so as to limit social desirability and reporting biases. Semi-structured interview guides (Tables 1 and 2) developed by the main study investigators included open-ended, exploratory questions and optional follow-up probing questions that allowed participants to introduce topics relevant to their experience. Domains of inquiry included usual HIV prevention practices before study participation; changes in sexual practices while in the study; acceptability and perceived feasibility of assigned dosing schedule; hypothetical preferences among the three schedules; and facilitators and barriers to adherence. Demographic information was collected at the main study enrollment visit via interviewer-administered questionnaire and sexual risk behavior via computer-assisted self-interview at the main study randomization visit; data were abstracted for sub-study participants.

**Table 1:** Focus group guide

Understanding of schedule
*Let’s begin by talking about the schedule that you were asked to follow for taking pills in this study*
1. What is your understanding of how you were asked to take pills in this study?
*Probe: What is your understanding of what you were being asked to do with the pills that you got in this study?*
Schedule fit to daily life (facilitators/challenges)
*Now I’d like to ask you about how difficult or easy it was for you to follow the recommended schedule for taking pills*
2. What would you say were the main things that made this recommended schedule work well for you or fit into your life?
3. What would you say were the main things that made this recommended schedule not work so well, or really fit well into your life? Were there times it was particularly hard to try to take the pills as recommended (prescribed)?
Feasibility/acceptability of assigned schedule
4. For your recommended schedule, [*define this for the group*] what would you say about how acceptable it would be to people in general?
*Probe: Who would it be good for? Who would it not fit well with?*
5. How reasonable is it to ask people to follow a pill-taking schedule that tells people to [*use schedule definition*]?
Schedule alterations
6. Many people find it difficult to take pills exactly as they are recommended, for lots of reasons. Sometimes people change how they take pills to better suit their lives. Are there any times you can think of when you changed the recommended schedule in this study to better fit your life? Would you share with the group how you did this?
*Probe to explore intentional changes to the schedule and any indications that people started and stopped study drug in relation to sexual activity*
Ideal schedule
7. What would your ideal schedule for taking these pills be? What would work best for you?
Preferences for other schedules
8. Other schedules being studied include [*provide details on schedules not assigned to this group and write each potential schedule on the flip chart: Daily* (*one tablet each day*)*; Twice Weekly‐Plus* (*take a tablet on two separate days of the week plus a tablet after each time you have sex*); *Before and After sex* (*take one tablet a day or so before sex and shortly after sex*)]. If all these schedules worked equally well to prevent HIV infection, which of these would you choose?
Perceptions of adherence support offered/provided
9. At each visit that you were given study drug, a counselor asked you about your experiences with the study pills. What were those discussions like?
10. How did those discussions affect your pill-taking, if at all?
11. What kind of changes would you recommend for those discussions?
12. Based on your experience, what would you recommend to help people who are not in a study take PrEP as their doctor recommends?
PrEP as part of/or in conflict with other prevention strategies
13. Let’s talk about what you normally do that may protect you from getting HIV. What kinds of things do you normally do?
14. When you started taking these tablets, did you notice a change in any of the other things that you normally would do to help to protect you from HIV?
*Probe for (1) strengthening of other prevention practices and/or (2) weakening of non‐PrEP prevention practices and what the group perceived the reasons for these changes were.*
Risk compensation
15. When you started taking these tablets, did you notice a change in your sexual behaviors?
*Probe for potential changes in number or types of partners, frequency of sex or engaging in different kinds of sex.*
16. Did taking the pills ever affect the way you thought about your risk of getting HIV? In what way(s)?
*Probe for preventive misconception‐ that use of the study medication provided greater benefits than reasonably expected.*
Social motivation/study connections
[*Just a couple of questions left.*]
17. Did anyone close to you know about your participation in this trial?
*Probe for partial disclosure of study procedures, e.g., did participants say that they were in a prep study but not that they were taking medication, or that they were on a specific dosing schedule*.
18. What kinds of things influenced your decision to tell people or not to tell people that you were in this study and/or that you were taking the study medication?
19. What would you say about any support you got from people important to you, the study team members you worked with during your clinic visits, or other participants you met during visits or as a part of your involvement in the study? Did you get any support from others? What would be an example of that?
Recommendations
20. Last question‐ Are there any observations or recommendations for the research team that you would like to offer that have not been discussed?

**Table 2:** In-depth interview guide

Understanding of schedule
1. What is your understanding of the schedule for pill taking that you were asked to follow?
Schedule fit to daily life (facilitators/challenges)
*Now I’d like to ask you about how difficult or easy it was for you to follow the recommended schedule for taking pills.*
2. What would you say were the main things that made this recommended schedule work well for you or fit into your life?
3. What would you say were the main things that made this recommended schedule not work so well, or really fit well into your life? Were there times it was particularly hard to take the pills as recommended (prescribed)?
Feasibility/acceptability of assigned schedule
4. For your recommended pill-taking schedule, what would you say about how acceptable it would be to people in general? Who do you think it would be good for? Who do you think it would **not** work well for?
5. How reasonable is it to ask people to follow a schedule that tells people to take pills on the schedule that you had *[use schedule definition]*?
Schedule alterations
6. Many people find it difficult to take pills exactly as they are recommended for lots of reasons. Sometimes people change how they take pills to better suit their lives. Are there any times you can think of where you changed the schedule to better fit your life?
*Probe for intentional changes to the schedule and any indications that people started and stopped study drug in relation to sexual activity.*
Ideal schedule
7. What would your ideal schedule for taking these pills be for you?
Preferences for other schedules
8. Other schedules being studied include Daily (one tablet each day); Twice Weekly‐Plus (take a tablet on two separate days of the week plus a tablet after each time you have sex); Before and After sex (take one tablet a day or so before sex and shortly after sex). If all these schedules for pill-taking worked as well as the others to prevent HIV infection, which of these would you choose?
Perceptions of adherence support offered/provided
9. Each visit that you were dispensed study drug, a counselor asked you about your experiences with the study tablets. What were those discussions like for you?
10. How did those discussions affect your pill-taking, if at all?
11. Would you recommend changes to those discussions?
12. Based on your experience, what would you recommend to help people who are not in a study take PrEP as it is recommended to them?
PrEP as part of/or in conflict with other prevention strategies
13. Let’s talk about what you normally do that can protect you from getting HIV. What kinds of things do you normally do (like condom use, talking about HIV status, getting HIV testing, limiting number of partners, and so on)?
*Probe for strengthening of other prevention practices and/or weakening of non‐PrEP prevention practices and what the respondent perceived the reasons for these changes were.*
Risk compensation
14. When you started taking these tablets, did you notice a change in your sexual behaviors?
*Probe for potential changes in number or types of partners, frequency of sex or engaging in different kinds of sex.*
15. Did taking the pills ever affect the way you thought about your risk of getting HIV? In what way(s)?
*Probe for preventive misconception‐ that use of the study medication provided greater benefits than reasonably expected.*
Social motivation/study connections
[*Just a couple of questions left.*]
16. Did anyone close to you know about your participation in this trial? What kinds of things influenced your decision to tell people or not to tell people that you were in this study and/or that you were taking the study medication?
17. What would you say about the kind of support you got from people important to you, the study team members you worked with during your clinic visits, or other participants you met during visits or as a part of your involvement in the study?
18. Did you get any support from others? Can you describe that support to me?
Recommendations
19. Last question‐ Are there any observations or recommendations for the research team that you would like to offer that have not been discussed?

#### Qualitative Data Analysis

Recorded FG and IDI were professionally transcribed, de-identified and entered into Dedoose, a web application for qualitative data analysis [33]. Three co-authors (JF, YH-M, ASL) reviewed and coded a subset of transcripts using a preliminary set of deductive codes derived from the guides, with additional codes identified and applied through grounded theory techniques of open coding and comparative analysis [34]. The results were scrutinized to eliminate redundant or imprecise codes, clarify code definitions, and construct a coding schema used to code the entire set of transcripts [35]. The complete set of coded excerpts was reviewed by the larger team for inconsistencies and discrepancies, which were resolved through an iterative process of discussion and re-coding [36]. A chart generated by Dedoose showing the presence or absence of codes in each transcript was scrutinized for indications of systematic differences in coding of FG and IDI. No systematic differences were observed (data not shown). Thematic analysis was then conducted on a data set of coded excerpts from both FG and IDI to identify themes [37] that characterized perceptions, motivations, and experiences related to PrEP use.

#### Ethical Review and Participant Compensation

The main HPTN 067/ADAPT study and the sub-study were reviewed and approved by the Columbia University Medical Center Institutional Review Board. All participants provided written informed consent to participate in the main and sub-studies. FG and IDI participants were compensated 50 dollars and roundtrip public transportation fare.

## Findings

As shown in Table 3, 37 of the 179 Harlem HPTN 067 study participants completed either a FG (N = 31) or an IDI (N = 6). Participants in the qualitative sub-study had a mean age of 34 years. One IDI participant identified as gender queer; the remaining 36 sub-study participants identified as MSM. Sub-study participants were 68% black, 11% white, and 8% Asian; 27% identified as ethnically Hispanic or Latino. Sixty-eight percent were unemployed. Sub-study participants reported a mean of 5.7 sexual partners (range 1–25) and a mean of 9.1 instances of condomless intercourse (range 0–55) in the 3 months preceding study randomization. Sub-study participants did not differ significantly from other main study participants in demographic characteristics and in reported sexual behavior (data not shown). Consistent with the approach of recruiting participants around the time of their final study follow-up visit, sub-study participants had higher retention in scheduled follow-up study visits as compared to other main study participants (mean retention 96.5% vs 82.9%; p < 0.001).

**Table 3:** Qualitative sub-study participant demographics

	FG (n = 31)	IDI (n = 6)	Total (n = 37)
Mean age	34	36	34
Gender identity			
MSM	31 (100%)	5 (83%)	36 (97%)
Gender queer	0 (0%)	1 (17%)	1 (3%)
Race			
Black	21 (68%)	4 (67%)	25 (68%)
White	3 (10%)	1 (17%)	4 (11%)
Asian	3 (10%)	0%	3 (8%)
Other^a^	4 (13%)	1 (17%)	5 (14%)
Hispanic ethnicity	9 (29%)	1 (17%)	10 (27%)
Unemployed	20 (65%)	5 (83%)	25 (68%)
Educational level			
Less than high school	7 (23%)	1 (17%)	8 (22%)
High school	9 (29%)	3 (50%)	12 (32%)
Technical training	2 (6%)	2 (33%)	4 (11%)
Some college	6 (19%)	0%	6 (16%)
College	7 (23%)	0%	7 (19%)

^a^Includes European (2), not defined (2), and Native American (1)

Qualitative analysis yielded three broad, interrelated themes: (1) PrEP is a significant advance for HIV prevention; (2) Non-daily dosing of PrEP is congruent with the episodic nature of HIV risk; and (3) Pervasive HIV-related stigma directed at PrEP use is a barrier to adherence.

### PrEP is a Significant Advance for HIV Prevention

Reflecting on how they protected themselves from HIV before using PrEP, participants described a combination of tactics that for most included at least some condom use, but also relied on their own assessment of the potential for HIV exposure that a given sexual partner represented and their ability to modify their own behavior accordingly. Some described this assessment as knowing a partner’s status, others as a nuanced evaluation of the risk represented by a potential partner.

“I’m not a condom user so what I did was I just try to keep my same partners…. And I know my partners’ status. So that was my safe sex. I knew their status.” FG Time-Driven Arm

“For me, if I was entertaining the thought of having sex with him, now comes the thing of getting to know your character and knowing your history to see if you’re promiscuous or you’re the cheating type, or the lying type.” FG Event-Driven Arm

Several participants reflected that their habitual approach to prevention before using PrEP was fallible, resting as it did on their ability to discern risk in their partners or resist the temptation to engage partners who might expose them to HIV.

“Everything that look good to you ain’t good for you. Everything that glitters ain’t gold.” IDI Daily Arm

“Sometimes I am not able to say no when I need to say no when I know it is the best thing for me to say no, sometimes.” IDI Time-Driven Arm

In comparison to their prior strategies, participants viewed PrEP as a groundbreaking advancement in HIV prevention and expressed deep appreciation for its potential to protect them from HIV. Words and phrases used to describe what PrEP meant to the participants ranged from scientific to popular culture references and even included a description of HIV as a childhood adversary. The descriptions served to emphasize that PrEP was a true departure from previous strategies for HIV prevention.

“The biggest discovery in the last century was antibiotics; man it’s saved so many lives. This is just as big as that to me.” FG Daily Arm

“It’s like having the Terminator by your side.” FG Event-Driven Arm

“I felt like I was protected. I was protected. Like that was my older brother and I was getting beat up by the bully, you know, at school.” IDI Time-Driven Arm

For some participants the alleviation of anxiety about HIV that PrEP afforded led to enriched sexual experiences.

“Maybe you’re a little bit more sexually liberated… aside from the condom it had to do with extra protection, so I would like – I would go a little extra hard, and if the condom broke, I wouldn’t be so scared.” FG Event-Driven Arm

“My sex life was reborn. Before, I wouldn’t have - but this had aroused – created a – it aroused a sense of curiosity, and I really rediscovered myself. I said, “Holy shit! Amen.” FG Daily Arm

“I’m doing things – I’m learning things that I should have been doing in my 20 s and 30’s…. [When] I was growing up – AIDS; boom…. Sex meant death.” IDI Daily Arm.

When asked how taking PrEP influenced their HIV prevention practices, participants frequently described becoming more aware of the need for prevention and adopting other protective behaviors during their time in the study.

“I think it changed my thought process a little bit. Like I thought about it a little more than I normally would have, like, ‘Am I really going to trust this pill to do this? Or should I just put this condom on, as well?’… Actually, I put more thought into it.” FG Daily Arm

“It made me reduce my partners by a huge amount…. Just taking the pills continually reminded me to protect myself from HIV. And one of the risks of having HIV is just having an excessive amount of sexual partners, and then having risky encounters.” FG Event-Driven Arm

Many participants explicitly rejected the notion that PrEP justified abandoning other prevention methods, and several emphasized the need to protect themselves against infections other than HIV.

“People just came up and asked me, like, ‘Hey, are you on the pill? Oh, good, so we can just like…’ – No, that’s not how it works for me.” FG Daily Arm

“This pill can help you but in conjunction with safer practices…. Because if you just take this one thing and not everything else it’s going to fall apart.” FG Event-Driven Arm

“It’s like I was bulletproof against the big package but the little packages could still slide up in there.” FG Time-Driven Arm

In contrast, a minority of participants acknowledged engaging in situations that they might have avoided without PrEP. Some explicitly connected their amplified risk behavior to the use of drugs or alcohol.

“I kind of turned it up a little bit, because I was on the medication, but again, I’d go back to the fact that I was actively using drugs, so I wasn’t in my right frame of mind, and of course, being on the medication made me think, “Hey, man, I’m protected.” FG Event-Driven Arm

One participant recalled reflecting on and correcting an initial willingness to forgo usual protective behaviors while taking PrEP.

“It made me practice safe sex less, a little bit less than I would normally…. I felt kind of impervious to it, to catching HIV, because of the pill. I really had to check myself about that, I really did.” FG Time-Driven Arm

### Non-daily Dosing of PrEP is Congruent with the Episodic Nature of HIV Risk

Participants across study arms generally expressed preferences for non-daily dosing schedules, both because non-daily dosing corresponded to patterns of sexual activity and because they believed side effects of the PrEP medication would be less with less frequent dosing.

“I’d rather do it when I have sex…. Like when I put on a condom, or the person I’m having sex with is putting on a condom, that’s something I have to do in that moment, and not every day.” FG Event-Driven Arm

“I know sometimes I’m just not going to have sex for a week or so, because I just really don’t feel like it. And taking the pill when you know you’re not going to have sex kind of sucks because you know that it can cause kidney damage and bone density troubles.” IDI Daily Arm

One participant who based his preference for dosing schedule on the demonstrated efficacy of daily PrEP acknowledged that a proven non-daily schedule would be preferable.

“I think if it will be like hundred percent proven, then the less the better, but until then, I will take it as much as they say.” FG Time-Driven Arm

Several participants reasoned that dosing frequency should be calibrated to the individual’s level of sexual activity.

“If people are having unprotected sex, then I think that they should take it every day. People that are… not really sexually active, I really think that twice a week would be a better suggestion for people like that. But people that are in a relationship and constantly having sex and intercourse and stuff like that, I think that they should take it every day.” IDI Time-Driven Arm

Some participants described acting on this rationale and adjusting their dose according to what they perceived as their level of risk.

“I did change my regimen a little bit when I found myself just being with a single partner for a bit over a month, so I wasn’t taking it nearly as much, because I didn’t feel I needed the prevention at the time.” FG Daily Arm

Despite the expressed preference for non-daily dosing, participants in non-daily dosing arms agreed that intermittent dosing presented adherence challenges. Remembering regular non-daily doses was difficult on the time-driven schedule; in both time-driven and event-driven arms forecasting sex in order to plan for dosing around sex was a challenge.

“I’m not going to lie, the whole remembering to take the pill thing, being as how I was set to take it twice a week, not like it was every day, it was a little hard to remember.” FG Time-Driven Arm

“You never really know, you might think that you’re not going to have sex and then you have sex. I actually didn’t have [any pills] on me because I wasn’t thinking about sex and then it happened, and I was like so far away from home that I wouldn’t have been able to achieve it right after sex, like I was supposed to.” FG Time-Driven Arm

Several participants observed that sex-dependent doses were challenging if sex was accompanied by drug or alcohol use.

“Only if I was like getting high I would probably forget to take my “before” pill. Then I would take my “after” pill, definitely.” IDI Event-Driven Arm

### Pervasive HIV-Related Stigma Directed at PrEP Use is a Barrier to Adherence

In contrast to the positive effects that PrEP had on sexual experiences and the generally heightened awareness of prevention that participants gained while in the study, experiencing or anticipating stigmatizing attitudes surrounding PrEP use was reported across study arms.

“Some people saw the pills I was taking and they thought I was sick. Oh he got HIV, because he taking Truvada.” IDI Daily Arm.

A few participants described contesting stigmatizing attitudes about PrEP in conversations with family and friends.

“[My mom] was like, “What does this mean? Are you trying to tell me that you have HIV?” And I said, “No, I’ll send an article – a news article – about what this is all about.” FG Daily Arm

However, the association with HIV infection was generally difficult to counter with facts about PrEP, as the following exchange among FG participants suggests.

“You say, ‘Yo, I’m on a PrEP regimen, dah–dah-dah.’ You can explain it. You can give them documents in front of them like this –”

“It’s not going to work.”

“- It’s not going to work. You know why? Because the only thing in their mind is AIDS.”

“Stigma.” FG Event-Driven Arm

Another type of stigma attached to PrEP related to the presumed promiscuity of PrEP users.

“One of my partners was like, ‘Whoa, whoa. What does this mean? What are you doing?’ In other words, indicating that just by taking the pill this means that I’m willy-nilly having unprotected sex.” FG Daily Arm

“When I was talking to guys online, there were a few of them that were just like – doesn’t matter if you’re on PrEP, I’d rather not have sex with you because I don’t know if PrEP works or you’re probably just like a big whore.” FG Time-Driven Arm

Some participants themselves acknowledged holding stigmatizing attitudes about PrEP use.

“For me, if someone just said so-and-so’s using PrEP and I knew what PrEP was at that time, I would question what is their HIV status…. [Or] they could just be very – really promiscuous.” FG Time-Driven Arm

One participant vividly imagined gossip spreading through his social circle and extending to the workplace, ultimately impacting his job.

“You could tell somebody [that you are taking PrEP] and no more than 10 minutes later… the whole neighborhood is saying, ‘Bob said he is taking them sex pills!’ His friend then told Tom and Tom done told Mary. Now look, when you get to work Monday they are telling you need a physical… and you saying, ‘What, boss? I just had a physical.’ The rumors started in Manhattan and you work in the Bronx. How do they get there?” FG Event-Driven Arm

To avoid negative reactions, participants were wary of disclosing their PrEP use to others.

“I confided in myself. I didn’t tell nobody, because I know people judge you; taking pills they think you’re sick, something wrong with you, so I just kept it to myself” FG Event-Driven Arm

In addition to stigmatizing reactions from family and friends about PrEP use, participants also described anticipating or experiencing stigma when taking PrEP tablets after a sexual encounter. Some participants assigned to non-daily dosing arms developed strategies to hide post-sex doses from their partner. One participant described hiding PrEP tablets in a mint box that he kept nearby during sexual encounters.

“You can have it [and tell your partner] - ‘One minute, hold on, let me go to the bathroom.’ Pop [the tablet in the bathroom], because… some people are not ready to just accept things. They live in - in a fantasy world where you don’t think it can happen to you.” FG Event-Driven Arm

Others described difficult interactions with partners who saw the tablets.

“I didn’t want to have them with me where the other person would find it because it was uncomfortable. Somebody did find them and I had to do some explaining, or it would have gotten into a big ugly fiasco.” FG Event-Driven Arm

“When I was having sex, I would take the pills and my partners would be like, ‘Why are you taking those pills?’… Sometimes it would never get to the intercourse part. It would just stop the night. They would be mad and leave.” IDI Time-Driven Arm

Similarly, some participants who were assigned to the daily dosing schedule saw the appeal of taking PrEP in conjunction with sex but anticipated that it would be difficult to disclose pill-taking to sexual partners.

“I really like the before sex [schedule]. I mean, but then again, it would have been awkward if the guy sees me taking my pill and he’s like – oh, what are you taking? And then I have to go and explain the pill and I don’t want to do that.” FG Daily Arm

## Discussion

The mainly black MSM participants in the Harlem HPTN067/ADAPT qualitative sub-study saw PrEP as a valuable addition to their existing HIV prevention strategies, which were based in part on their ability to discern risk in their partners and adjust their behavior accordingly. Participants commonly described a heightened awareness of prevention while taking PrEP and adopting additional protective behaviors, perhaps in part due to the regular counseling and HIV testing they received as part of study participation. At the same time, a minority of participants reported increased willingness to engage in sexual encounters that might put them at risk for HIV, similar to the reductions in condom use among some PrEP patients that has been noted in clinical practice settings [38]. The experience of recognizing and correcting increased risk behavior while on PrEP has been identified in other qualitative studies of PrEP use [39], highlighting that individual prevention practices are responsive to both perceptions of situation-specific risk and the internalization of support for practicing prevention behaviors [40, 41].

In keeping with a situation-specific perspective on risk for HIV, Harlem sub-study participants frequently conceptualized adherence to PrEP as situational. They related the need to take PrEP to periods of condomless sex, number of current sexual partners, and other factors contributing to HIV risk such as substance use during sexual encounters. This perspective on taking PrEP is consistent with the emergent paradigm of ‘prevention-effective’ adherence to PrEP, in which the success of PrEP rests on accurately recognizing the potential for exposure to HIV and utilizing other prevention tools appropriately, in addition to achieving adequate adherence to PrEP medication [42]. The findings presented here suggest that dynamic approaches to PrEP aligned with levels of exposure to HIV, including non-daily dosing schedules, would be acceptable to potential users. However, Harlem participants noted challenges to such approaches, including establishing routines for intermittent pill taking, forecasting sexual encounters, having medication available proximate to sex, and managing sexual partners’ attitudes about PrEP. Strategies for helping PrEP users recognize patterns of situational risk and adopt appropriate prevention practices such as limiting the number of sexual partners and ensuring that condoms are available may be a valuable addition to counseling on adherence to PrEP [42]; further implementation research on the optimal components of support for PrEP is needed.

The most vividly-described challenges that participants reported experiencing with sex-dependent dosing involved hiding PrEP use from sexual partners and dealing with partners’ negative response if participants disclosed PrEP use. Paradoxically, even as participants became more aware of their need for prevention and strengthened their prevention-related practices, they reported experiencing or anticipating stigmatizing attitudes from family, friends and sexual partners who interpreted the use of a recognized anti-HIV medication as an indication of being HIV-infected or associated PrEP use with promiscuity and exceptionally high risk for HIV.

Stigma related to both HIV and sexual promiscuity is a recognized barrier to PrEP uptake and use [17, 23, 43, 44]. Research into attitudes on PrEP use among the general population in the US documents that stigma regarding PrEP is influenced by existing frameworks of stigma, so that PrEP use among stigmatized groups, notably black MSM, is viewed more negatively than PrEP use among the general population [45]. Our study adds a layer of complexity to our understanding of how stigma impacts on PrEP use, highlighting the challenge stigma may present for sex-dependent PrEP dosing. This finding is relevant to efforts to extend the findings of the ANRS IPERGAY study, in which a dosing strategy of two tablets of FTC/TDF two to 24 h before sexual intercourse and a third and fourth tablet at 24- and 48-h intervals after the first dose demonstrated 86% relative reduction in risk of HIV infection among MSM and TGW in a placebo controlled trial [16] and 97% relative risk reduction in the follow-on open-label. Further exploration of how stigma related to promiscuity and HIV treatment intersects with HIV prevention strategies and how it can be mitigated at the social and interpersonal level is critical [43, 44, 46, 47].

### Limitations

These findings are subject to several limitations. The sub-study enrolled a convenience and purposive sample of mostly black MSM in New York City that is not generalizable to other populations in other areas. Although staff endeavored to reach study participants who had missed study visits to invite them to participate in the qualitative study, sub-study participants were significantly more likely to be retained in study follow-up visits than were other main study participants. Participants who were able to be reached and willing to participate in qualitative FG and IDI may be different than those who could not be reached or were unwilling to participate in the qualitative research. The group setting may have inhibited FG participants from voicing some opinions or sharing some experiences. Finally, the sub-study recruitment procedures were not designed to ensure participation by TGW participants in the main study and the sub-study sample included no TGW. TGW are disproportionately impacted by HIV and evidence suggests that they may experience elevated challenges adhering to PrEP [48]; more research into the experience of this highly vulnerable group is crucial.

### Strengths

Our sub-study engaged mostly black MSM, a group highly impacted by HIV in the US [49]. They lived in an area with high HIV prevalence and were largely unemployed, a factor associated with likelihood of HIV infection among black MSM [50]. Thus, they represent a group likely to benefit from the successful implementation of PrEP in the US [51]. Unlike most qualitative studies conducted outside of PrEP efficacy trials, participants had completed several months of open label PrEP and the data reflect that experience rather than anticipated or hypothetical perceptions, preferences, barriers and facilitators.

## Conclusions

As PrEP uptake in the US grows, the need for interventions to support its effective use becomes ever more urgent. The qualitative data presented here highlight critical areas of needed support, including integrating PrEP into other prevention strategies, adherence interventions for dosing schedules that are congruent with patterns of sexual activity and intensifying efforts to counter PrEP-related stigma. The findings are particularly relevant in characterizing PrEP-using experiences of Black MSM—a crucial population for HIV prevention efforts in the US.
